# Development of a Model Predicting the Outcome of *In Vitro* Fertilization Cycles by a Robust Decision Tree Method

**DOI:** 10.3389/fendo.2022.877518

**Published:** 2022-08-24

**Authors:** Kaiyou Fu, Yanrui Li, Houyi Lv, Wei Wu, Jianyuan Song, Jian Xu

**Affiliations:** ^1^ First Affiliated Hospital, School of Medicine, Zhejiang University, Hangzhou, China; ^2^ School of Control Science and Engineering, Zhejiang University, Hangzhou, China; ^3^ Women’s Hospital, School of Medicine, Zhejiang University, Hangzhou, China; ^4^ Fourth Affiliated Hospital, School of Medicine, Zhejiang University, Yiwu, China

**Keywords:** *in-vitro* fertilization, artificial intelligence, prediction model, feature discretization, clinical pregnancy

## Abstract

**Introduction:**

Infertility is a worldwide problem. To evaluate the outcome of *in vitro* fertilization (IVF) treatment for infertility, many indicators need to be considered and the relation among indicators need to be studied.

**Objectives:**

To construct an IVF predicting model by a robust decision tree method and find important factors and their interrelation.

**Methods:**

IVF and intracytoplasmic sperm injection (ICSI) cycles between January 2010 and December 2020 in a women’s hospital were collected. Comprehensive evaluation and examination of patients, specific therapy strategy and the outcome of treatment were recorded. Variables were selected through the significance of 1-way analysis between the clinical pregnant group and the nonpregnant group and then were discretized. Then, gradient boosting decision tree (GBDT) was used to construct the model to compute the score for predicting the rate of clinical pregnancy.

**Result:**

Thirty-eight variables with significant difference were selected for binning and thirty of them in which the pregnancy rate varied in different categories were chosen to construct the model. The final score computed by model predicted the clinical pregnancy rate well with the Area Under Curve (AUC) value achieving 0.704 and the consistency reaching 98.1%. Number of two-pronuclear embryo (2PN), age of women, AMH level, number of oocytes retrieved and endometrial thickness were important factors related to IVF outcome. Moreover, some interrelations among factors were found from model, which may assist clinicians in making decisions.

**Conclusion:**

This study constructed a model predicting the outcome of IVF cycles through a robust decision tree method and achieved satisfactory prediction performance. Important factors related to IVF outcome and some interrelations among factors were found.

## 1 Introduction

Infertility is a worldwide problem that affects tens of millions of families. It is estimated that 1 in 6 couples in the world experiences infertility (1). The development of assisted reproductive technology (ART) has brought hope to couples with infertility. There were over 280,000 ART cycles and over 70,000 liveborn infants in the US according to the US Centers for Disease Control and Prevention 2017 Fertility Clinic Success Rates Report (2). Also, China has made great efforts to treat infertility that the total number of cycles of ART has exceeded 1 million and the number of infants born has exceeded 300,000 per year in China (3). However, despite the big number of ART cycles, clinical pregnancy rate per embryo transfer was as low as 30% (4). There are many factors identified to play important roles in the outcome of IVF, such as age, body mass index (BMI), hormone levels and ovarian reserve capacity (5–9). These various factors make it complex to evaluate the outcome of IVF cycles before implantation. It is also a financial burden for patients with infertility to perform IVF cycles. Therefore, there is a pressing motivation to improve the way in which those factors are integrated to predict the outcome of IVF cycles.

Nowadays, data-driven analysis based on machine learning has been more and more applied in the field of medical problems where the success mainly depends on feature engineering and model selection. Feature-engineering, including feature selection and feature extraction, can better mine information from the original data and improve the data quality which is especially indispensable for multi-variable data (10). Among them, the binning method is used for discretizing continuous variables which has advantage in increasing stability and robustness of data by avoiding the fluctuation caused by the meaningless fluctuation of the feature and avoiding the influence of extreme values (11). Besides, discretization can introduce the nonlinear characteristics of variables into the linear model so as to improve the expression ability and increase the fitness of the model. For instance, metagenomic binning has been widely used in metagenomic research, which aims to classify the contigs obtained from different organisms according to the species (12, 13). For model selection, the complexity and accuracy of the model need to be considered based on the characteristics of the data. For medical data, a simple logistic regression cannot process nonlinear data, although it has good interpretability. On the contrary, the complex deep learning model with high accuracy is hard to be applied in practice due to its unexplainable characteristics and the demand for large data samples. The decision tree methods, especially the Gradient Boosting Decision Tree (GBDT), which balances the accuracy and complexity are more suitable for our problem (14). Therefore, the aim of this study was to construct an IVF predicting model to estimate the chance of success implantation by GBDT based on discretized medical variables and to determine valuable factors affecting the outcome in IVF treatment.

## 2 Materials and Methods

### 2.1 Sample

IVF and ICSI cycles between January 2010 and December 2020 in Women’s Hospital School of Medicine Zhejiang University were screened. Patients with all causes of infertility were included. Exclusion criteria were 1) patients with egg or sperm donor; 2) patients with preimplantation genetic diagnosis (PGT-M) or screening (PGT-A); 3) patients with frozen embryo transfer; 4) patients without treatment outcomes; 5) incorrect information or important data missing in the database. A total of 49413 cycles were collected, and 37062 were included in our analysis. Comprehensiv e diagnostic evaluation of infertility of patients, their specific therapy strategy and the outcome of treatment were recorded. Samples were divided into clinical pregnant group and nonpregnant group according to whether the patient has clinical pregnancy which needs evidence of both HCG and ultrasonography after *in vitro* fertilization and embryo transfer. The study was approved by the Institutional Review Board at Zhejiang University (IRB-2020 0235-R) and was carried out in accordance with the Helsinki Declaration.

### 2.2 Study Design

**Figure 1:**
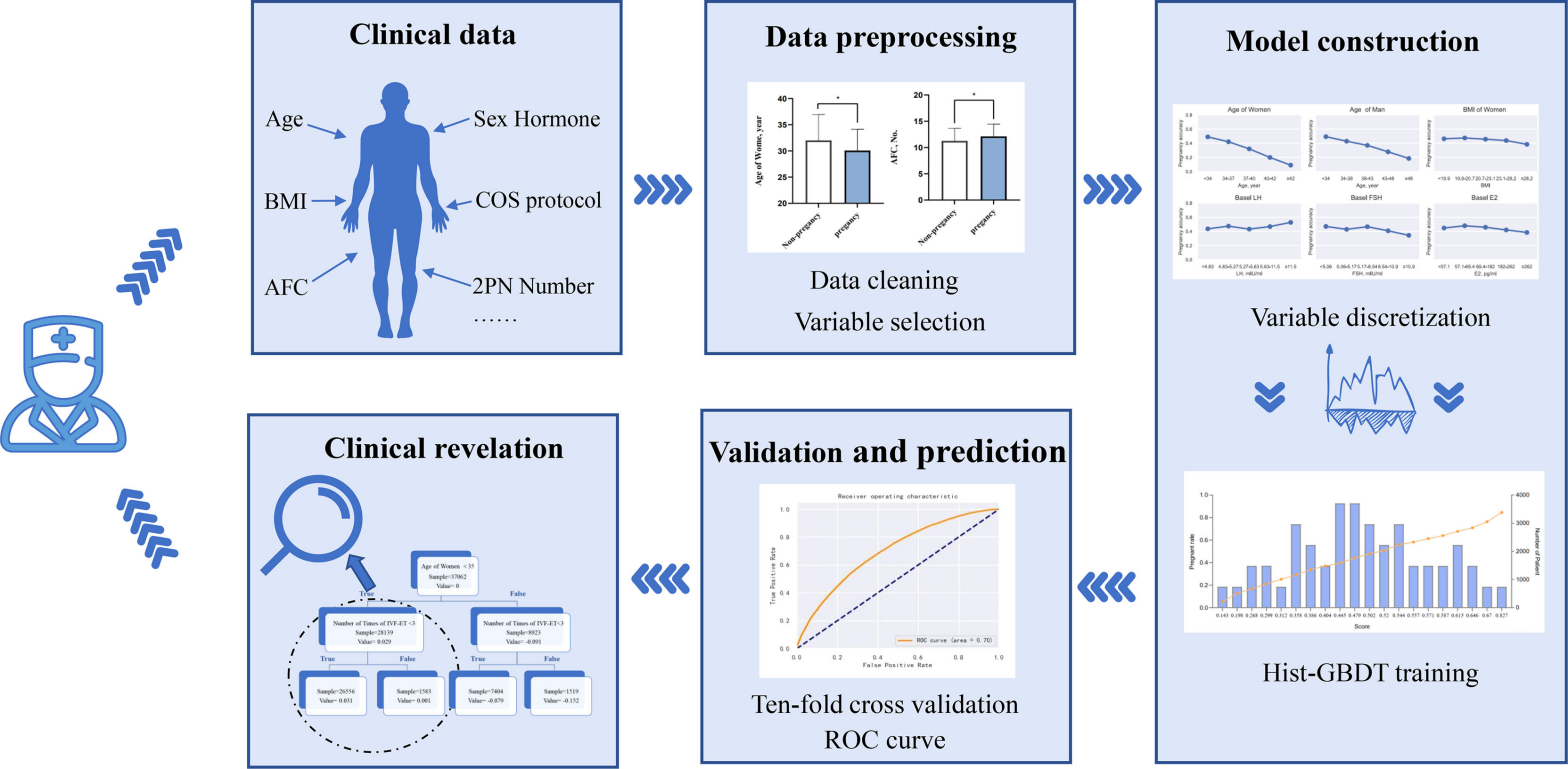
Flow chart of the study This figure described the flow sheet of the study. GBDT (Gradient Boosting Decision Tree).

The flowchart of this study is shown in **Figure 1**. First, obvious outliers were removed according to the possible ranges of indicators. Second, Indicators were selected through the significance of 1-way analysis between the clinical pregnant group and the nonpregnant group. Third, the selected indicators were discretized. Then, a complete model was constructed by GBDT and the performance of the model was validated. Finally, important factors related to IVF outcome and some interrelations among indicators with clinical meaning in model were found.

### 2.3 Variable Selection by One-way Analysis

Between the clinical pregnant group and nonpregnant group, the basic characteristics of infertile couples (including age, BMI, type of the infertility, history of pregnancy and delivery, causes and duration of infertility, basal FSH level, basal LH level, and antral follicles count, etc), the factors in controlled ovarian stimulation (COS) procedures (including COS protocol, time of Gonadotropin days and Gonadotropin dosages, number of large follicles, the number of retrieved oocytes and serum hormone level of HCG trigger day, etc) and the factors during the embryo transfer procedures (including the number of bipronuclear, embryo culture time, number of embryo transferred, endometrial thickness and endometrial type, etc) correlating with the outcome of clinical pregnancy were analyzed. A total of 47 variables were included (**Appendix Table 1**). After one-way analysis of variance, only variables with p value < 0.05 were selected to the binning procedure.

### 2.4 Binning Procedure

As mentioned before, the binning procedure is helpful in increasing stability and robustness of model and the discretized data can show the relationship between variables and outcome clearly. In our work, factors were discretized by chi-merge algorithm. The chi-merge algorithm is a bottom-up discretization method which bins the variable by merging the adjacent intervals with the smallest chi-square value. The specific steps were given in **Appendix 1** in the Supplement.

### 2.5 Model Construction

A decision tree is a flowchart-like structure in which each internal node represents a ‘test’ on an input variable and each sample will go through one path from root to leaf to get a prediction. GBDT is an ensemble machine learning technique for regression and classification problems, which uses decision trees as weak prediction model and ensembles them to produce a strong prediction model. Models are built in a stage-wise fashion and generalized by the optimization of an arbitrary differentiable loss function. Discretizing the continuous input variables to unique values can dramatically accelerate the training process, which is called histogram-based gradient boosting decision tree (hist-GBDT) (15). Because of the binning procedure, our work could be regarded as a specific hist-GBDT form. Details about GBDT were given in appendix 3 in the supplement.

### 2.6 Model Validation

Patients were scored according to the model. Receiver operator characteristic (ROC) curve was constructed and the area under ROC curve (AUC) value was computed to validate the performance of pregnancy prediction. Also, ten-fold cross validation which is a method used for verifying the stability of the model was performed. The specific steps were given in Appendix 3 in the Supplement.

### 2.7 Statistical Analysis

In the process of data analysis, 1-way analysis of variance of the variables was performed by R statistical software version 3.6.2 with package “multcomp 1.4”. Binning, model construction and validation were conducted by Python 3.7.1 with package “numpy 1.21, scipy 1.6, and pandas 1.2”. Two-tailed tests and p values <0.05 for significance were used.

## 3 Results

### 3.1 Variable Screening

A total of 37062 cycles were included and divided into two groups according to whether the patients were clinical pregnant or not after *in vitro* fertilization and embryo transfer. Among them, 16823 samples were in the pregnant group with an average age of 30.73 years while 20239 samples were in the non-pregnant group with an average age of 32.01 years. Detailed features of two groups were shown in **Supplementary Table 1**.

Thirty-eight variables were found to have significant differences between the clinical pregnant group and the nonpregnant group by one-way analysis of variance, including demographic characteristics like age of couples and duration of infertility. Also, ovarian reserve capacity indicators such as antral follicle count (AFC) and anti-Mullerian hormone level (AMH) were also different in two groups. Besides, some factors during the IVF-ET procedure showed great discrepancy, for instance, treatment strategy, number of oocytes retrieved and number of 2PN. For example, compared with the nonpregnant group, the pregnant group had lower age (mean [SD]: 30.73 [4.08] years vs 32.01 [4.95] years; P <.01), higher AFC (12.12 [2.33] vs 11.25 [2.40]; P <.01), and greater number of oocytes retrieved as well as 2PN (11.88 [6.51] vs 10.64 [6.60]; 6.69 [4.21] vs 5.70 [4.15]; P <.01). Among 38 indicators with significant differences between the two groups, 6 variables were categorical variables, the other 32 variables were continuous and were selected for binning.

### 3.2 Binning Procedure and Variable Selection

**Figure 2:**
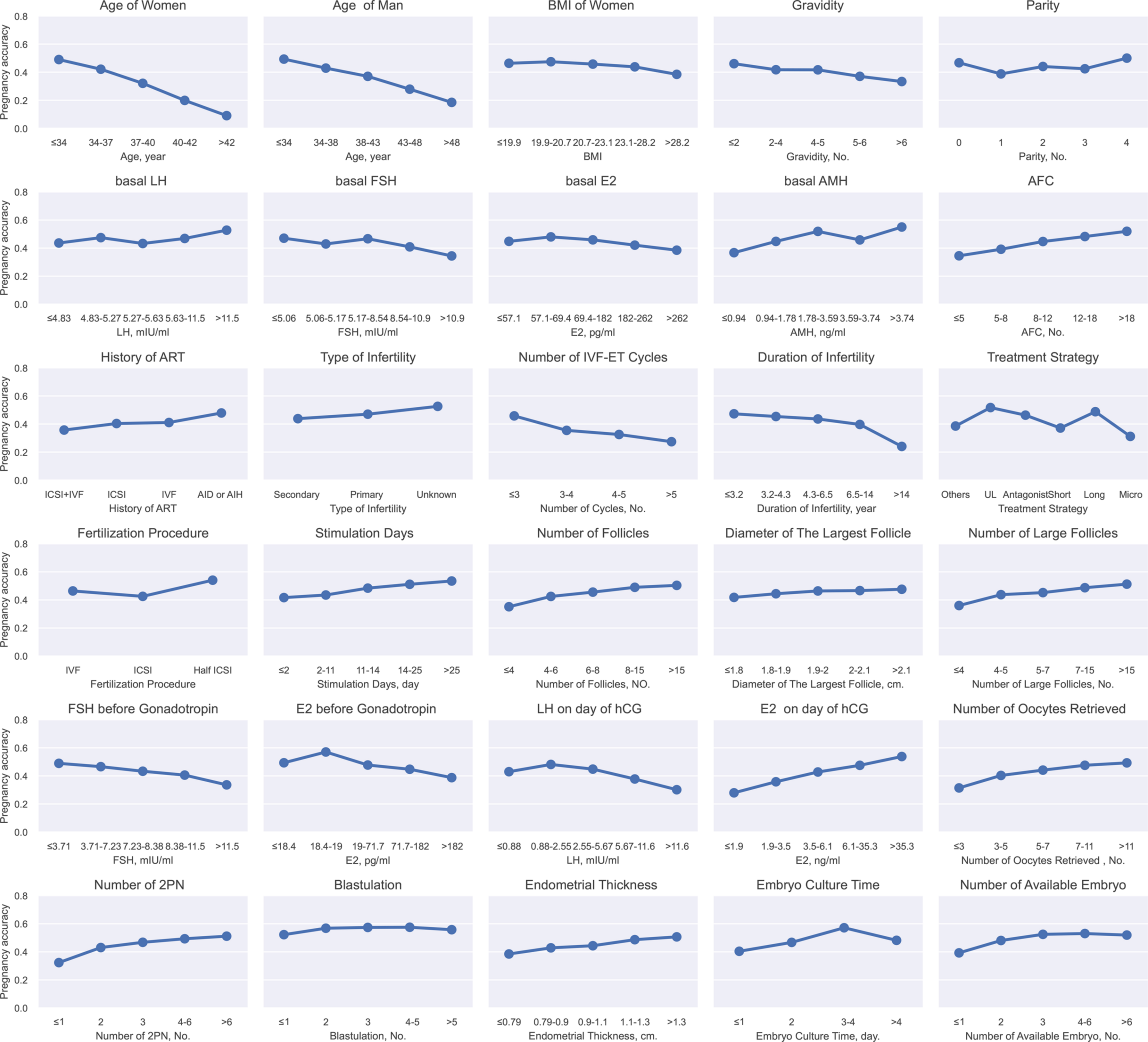
Continuous variables after binning and categorical variables for GBDT. This figure described the continuous variables after binning and categorical variables for GBDT. The X-axis represented the different grades of continues variable after binning or categorical variables. The Y-axis represented pregnancy rate. BMI (body mass index), LH (luteinizing hormone), FSH (follicle-stimulating hormone), E2 (estradiol), AMH (anti-Mullerian hormone), AFC (Antral follicle count), IVF (*in vitro* fertilization), ICSI (intracytoplasmic sperm injection), AI (artificial insemination), UL (ultra-long), Micro (microstimulation), hCG (human choriogonadotropin).

During the binning, continuous variables were discretized and transformed to five grades by the chi-merge algorithm. For example, with the increasing age, the clinical pregnancy rate dropped from 49.0% to 9.0%. Besides, there were some variables that didn’t show a linear correlation to the pregnancy rate, such as the embryo culture time. The success rate reached 57.1% at 3 days but was lower when the embryo culture time was less than 3 days or exceeded 4 days, which proved the necessity of using GBDT, a nonlinear model. Finally, 30 variables whose pregnancy rate varied in different categories were selected for GBDT-based model construction by the criterion “maximum clinical pregnancy rate difference between groups > 5%”, including 5 categorical variables and 25 binned continuous variables (**Figure 2**).

### 3.3 Model Construction

**Figure 3:**
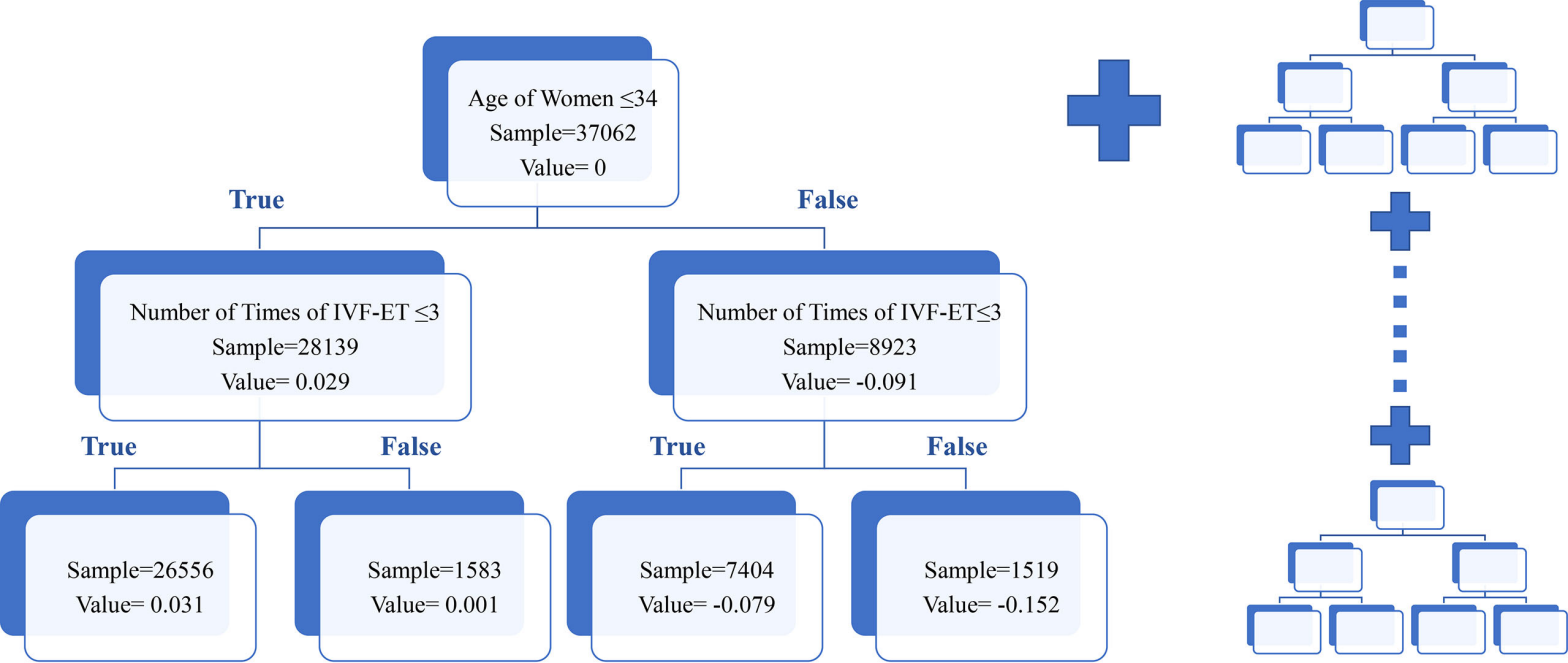
Schematic diagram of the GBDT model. This figure described the schematic diagram of the GBDT model. For convenience, only one decision tree was displayed. Patients were assigned to different categories according to the decision criteria. The value represented the pregnant possibility in different categories in certain tree. The total score was the sum value of all trees in model.

The aforementioned variables were used to build the final comprehensive evaluation model and a score was computed to predict the rate of clinical pregnancy by GBDT algorithm. **Figure 3** showed the internal construction of our model. GBDT was the sum of many similar decision trees. The left side of the figure 3 was one specific tree. Each sample (patient) will reach a leaf node in each decision tree and get a corresponding value. The score of a sample which predicted the success of clinical pregnancy was calculated by adding all values of all trees.

**Figure 4:**
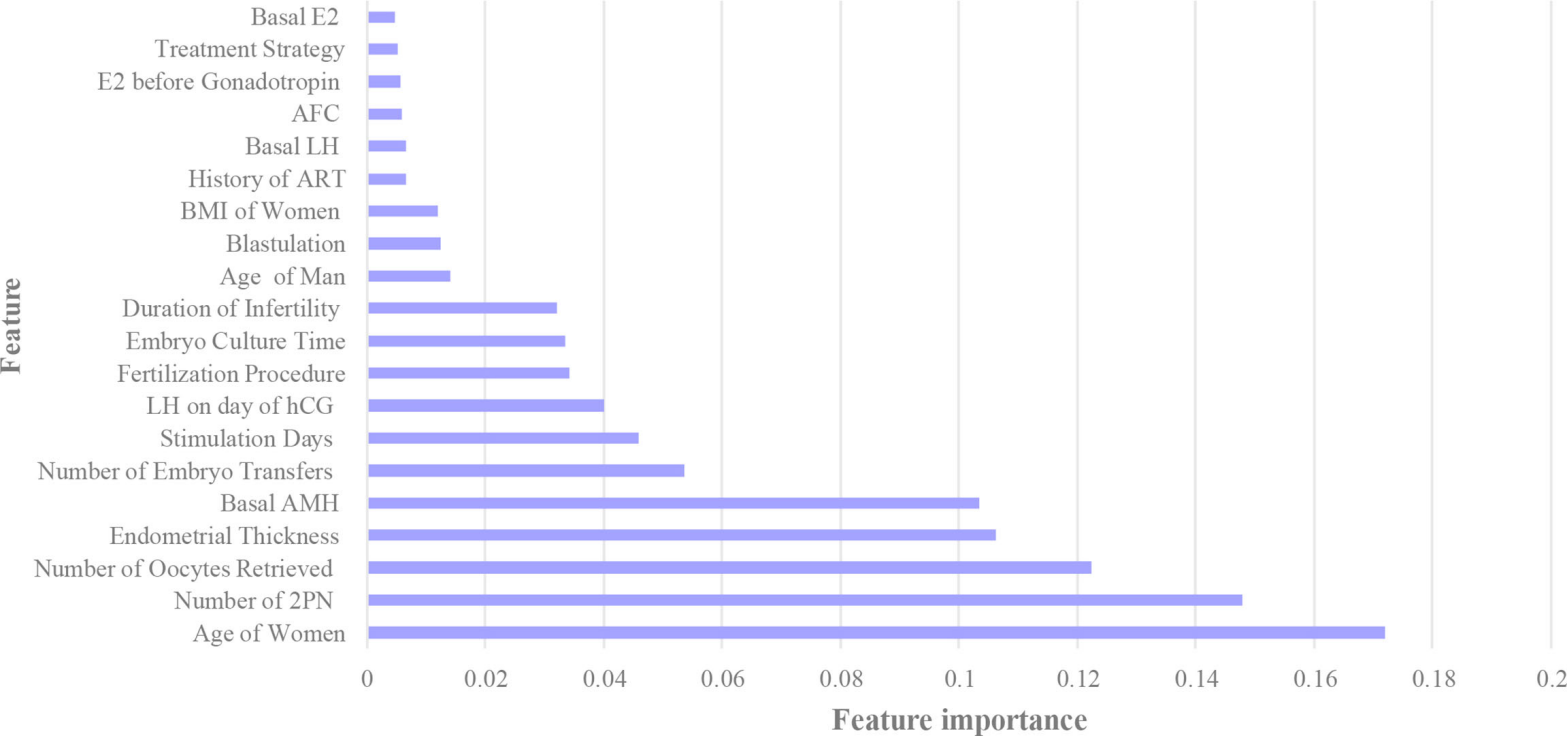
Importance of features. This figure described the importance of features. The relative importance value of top twenty features in Gradient Boosting Decision Trees were shown.

Besides, the importance of features was evaluated by the model, as displayed in **Figure 4**. Age of women, number of 2PN, AMH level, number of oocytes retrieved and endometrial thickness were the most important variables related to the outcome of the cycle.

**Figure 5:**
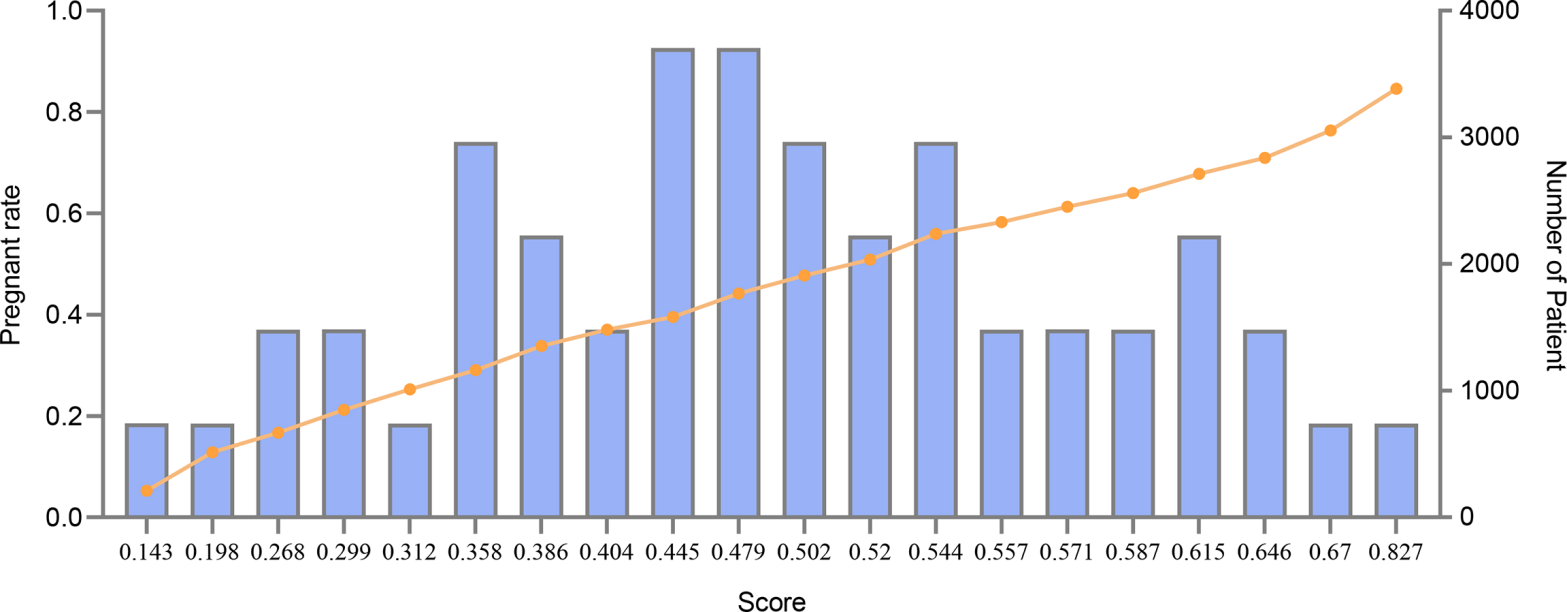
Pregnancy rate and total scores of patients. This figure described the association between pregnancy rate and total scores of patients. The X-axis represented the score. The line represented pregnancy rate. The bar represented the number of patients.

The association between the clinical pregnancy rate and the final score is shown in **Figure 5**. The score was calculated by our model and represented the predicted pregnancy rate, while the left y-ordinate in **Figure 5** was the actual pregnancy rate of the sample. The yellow line showed that there was a positive correlation between the score and the pregnancy rate, indicating our model was effective. The clinical pregnancy rate reached 84.6% for whose score was higher than 0.83 while the clinical pregnancy rate was only 12.8% whose score was lower than 0.2. Also, sixty percent of patients scored between 0.4 and 0.6, indicating the distribution of the score was in accord with the actual situation. Besides, the number of patients in each score section was more than 700, ensuring clinical pregnancy rate was stable rather than an extremum averaged by minor sample.

### 3.4 Model Validation

**Figure 6:**
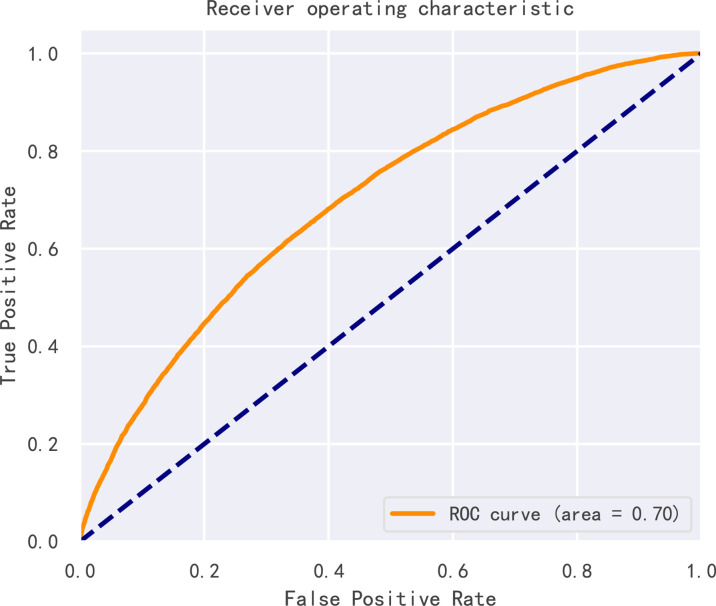
Receiver operating characteristic curve (ROC) of the model. Receiver operating characteristic curve (ROC) of the model.

By dividing patients into high possibility of clinical pregnancy and low possibility of clinical pregnancy using different thresholds of scores, ROC curve was constructed and the AUC value was 0.704 (95% CI, 0.699-0.709), as shown in **Figure 6**, proving that our model had good prediction performance. Also, ten-fold cross validation showed that the classification consistency of the model reached 98.1% (95% CI, 0.973-0.988) so that our model had excellent stability.

### 3.5 Clinical Revelation in Decision Tree

Aside from knowing the association between the single variable and the clinical pregnancy rate, the decision tree analysis also provided us with information about interaction among variables which may help in the clinic. For example, from the tree in **Figure 3**, patients aged over 35 years old may get harmed from repeatedly performing IVF that with the increase of the numbers of cycles, the value reflected clinical pregnancy rate dropped a lot. However, patients aged younger than 35 years old may be less affected since the value hardly changed. More specifically, the original data revealed when the number of cycles increased from 1 to more than 5, the clinical pregnancy rate decreased only 6.6% in the younger group while the rate declined by 13.6% in the older group (**Supplementary Table 2**). Similarly, we found some interesting discoveries from other trees. For instance, we found that women with lower AMH may benefit more from the short strategy, which was consistent with the consensus achieved by expertise (**Supplementary Table 3**). Those findings may assist clinicians in making an efficient and accurate judgment on the condition of patients with infertility.

## 4 Discussion

Infertility has attracted unprecedented attention worldwide nowadays. Despite that IVF and ICSI are the recommended and effective treatments for infertile couples, nearly half of couples who undergo IVF remain childless, even after multiple treatment cycles (16). Since treatment is expensive and invasive, couples with fertility problems need to undergo a complete assessment combined with various factors and be informed about their chances to succeed to make the decision. Over the past decades, many IVF prediction models have been developed to evaluate individual outcome of treatment but few of them were clinical practical due to their poor predictive ability and simple statistical method (17).

Machine learning which provides the sight to interpret data and construct prediction models has been increasingly applied to clinical issues, especially in complex systems with multi-variable (18–20). Recently, machine learning algorithms have been used in the reproductive field, for example, Khosravi et al. managed to select the highest quality embryos which may lead to a viable pregnancy by machine learning algorithms using visual images of the embryos (21). In terms of predicting IVF outcomes, it was suggested that machine learning algorithms based on age, BMI, and clinical data have an advantage over classic logistic regression and several models have been constructed by different algorithms (22–24). However, their models’ qualities were limited by small sample sizes, inadequate statistical methodology and lack of internal or external validation (17).

For the first time, we built a model predicting the outcome of IVF cycles innovatively combining GBDT and discretization in a large sample. After selecting the variables with significant difference between the clinical pregnant group and the nonpregnant group, continuous variables were transformed into five grades and assigned with separate weights by the binning algorithm. Clinical pregnancy rate varied in different categories after discretization, supporting that binning was an appropriate and excellent method to process clinical data with broad ranges and interference of fluctuation. Then the model was constructed by GBDT, a novel machine learning algorithm and by which the importance of features and total score evaluating the success of pregnancy were determined. The association between the pregnancy rate and the final score was strong that their trends were highly consistent. The clinical pregnancy rate reached 83.9% for those whose score was higher than 0.8 while the clinical pregnancy rate was only 11.2% for those whose score was lower than 0.2. Moreover, the distribution of the score was similar to normal distribution which testified our model reflected the actual situation. The AUC value of the model was 0.704, indicating that our model had a good performance. Also, ten-fold cross validation showed that the classification consistency of the model reached 98.1% which means our model construction method also had excellent stability.

The five most important features related to the outcome of treatment were age of women, number of 2PN, AMH level, number of oocytes retrieved and endometrial thickness. Female age was one of the strongest factors in predicting pregnancy chances after IVF and was identified by nearly all studies as an important predictor (25, 26). The underlying biological explanation included the diminished ovarian reserve, the decrease in both quantity and quality of oocytes with aging (27). In our study, women younger than 34 years old had the highest possibility to be pregnant with the total rate of 49.0%. Our study also showed that the number of 2PN is a significant predictor. Although Both 1PN- and 0PN-derived blastocysts can be used for embryo transfer, 2PN blastocysts indicated greater chance of success (28). The positive correlation between AMH level and the pregnancy rate found in our study was consistent with prior studies (29, 30). AMH represents the ovarian follicular pool and has been used as a marker of ovarian reserve for a long time. Besides, a positive association between increasing number of oocytes retrieved and pregnancy chances after IVF was reported by many researchers (26). We found that once the number of oocytes exceeded five, the clinical pregnancy rate reached 40% in our cohort. Similar to other research which defined 7mm as the cut-off of endometrial thickness, we found females with endometrial thickness less than 8mm may have negative outcome after IVF (31). Apart from above variables, others such as basal FSH, method of fertilization (IVF or ICSI) and number of embryos transferred were also related to IVF outcomes (32).

Our model also provided us with some information about interaction among indicators which may help in clinic. From specific decision trees in model, we concluded many interesting discoveries. For example, our study revealed multiple IVF cycles may cause harm to women over 35 years old but hardly influenced women younger than 35. Thus, clinicians need to be more cautious when treating patients aged over 35 because the failure of one cycle may be accumulated and affect the next cycle. Also, for women whose age exceeded 35, the number of oocytes retrieved had a great effect on the clinical pregnancy rate which increased a lot with the rising number of oocytes. However, the impact disappeared in young patients. It reminded us that finding ways to improve the number of oocytes retrieved to increase the clinical pregnancy rate may be a good choice for older women but may be less effective for the young. Besides, women with lower levels of AMH may benefit more from the short strategy when choosing COS protocol, which was consistent with the consensus reached by expertise. Those clinical revelations concluded in our model may in turn assist clinicians in making decisions on the complex condition of patients with infertility.

Our research provided a new method for IVF data processing and achieved satisfactory prediction performance. This approach can be applied to various clinical problems with multiple variables where classic statistics and analysis methods may not work. However, our study had several shortages. Firstly, although the sample size was large, there were missing data in certain variables, which may cover some discoveries. Secondly, despite that the result of ROC and ten-fold cross validation showed good internal validation, our study was absent of external validation due to the heterogeneity of data in different clinical centers. Also, there may be regional and population limitations in applying our model. The binning was based on the sampled data with specific ethnic and characteristics distribution which were not universal in the world. Therefore, when performing external validation, our binning and model may need to be adjusted if the distribution of samples’ characteristics change significantly. Thirdly, our model was suitable for patients with satisfactory uterine conditions who are ready for an IVF cycle, the effects of uterine abnormalities were not involved in this paper. In the future, we will continue to work on the practice of the model and to investigate the indicators’ relationship with IVF outcome to better guide the clinical treatment. Furthermore, we will apply our method to specific type of infertility (for example, unexplained infertility) to explore the impact of variables and relationship between variables on IVF outcome.

## 5 Conclusion

This study constructed a model predicting the outcome of IVF cycles combining binning and GBDT algorithm and achieved satisfactory prediction performance. Number of 2PN, age of women, AMH level, number of oocytes retrieved and endometrial thickness were important factors in relation to IVF outcome and some interactions between factors were found.

## Data Availability Statement

The original contributions presented in the study are included in the article/**Supplementary Material**. Further inquiries can be directed to the corresponding author.

## Ethics Statement

The studies involving human participants were reviewed and approved by Medical Ethics Committee of Women’s Hospital, Zhejiang University. The ethics committee waived the requirement of written informed consent for participation.

## Author Contributions

KYF: Methodology, Data curation, Writing-Original draft preparation. YRL: Software, Factor model construction, Writing. HYL: Data curation. WW: Investigation. JYS: Validation. JX: Resources, Supervision. All authors contributed to the article and approved the submitted version.

## Funding

This study was funded by National Key Research and Development Program of China (grant no. 2019YFC0121000).

## Conflict of Interest

The authors declare that the research was conducted in the absence of any commercial or financial relationships that could be construed as a potential conflict of interest.

## Publisher’s Note

All claims expressed in this article are solely those of the authors and do not necessarily represent those of their affiliated organizations, or those of the publisher, the editors and the reviewers. Any product that may be evaluated in this article, or claim that may be made by its manufacturer, is not guaranteed or endorsed by the publisher.
