# Supplementary material for: Development of a Model Predicting the Outcome of In Vitro Fertilization Cycles by a Robust Decision Tree Method
Source: Front Endocrinol (Lausanne). 2022 Aug 24;13:877518. doi: 10.3389/fendo.2022.877518 (PMC9449728; doi:10.3389/fendo.2022.877518)
Supplement: Supplementary file 1 [file Table_1.docx]

# Supplemental Online Content

Appendix 1. Chi-merge Variable Discretization Algorithm

Appendix 2. The theory of GBDT

Appendix 3. 10-fold Cross Validation

Table 1. Comparison Between Pregnant Group and the Non-pregnant Group

Table 2. The interrelation between age of women and number of cycles in model

Table 3. The interrelation between AMH and COS strategy in model

#### 1 Appendix 1. Chi-merge Variable Discretization Algorithm

The chi-merge algorithm are computed under the following pseudo code:

| **Algorithm 1:** Chi-merge discretization algorithm | |
| --- | --- |
| Data: the countious value of variables $X(x_{1},x_{2},\cdots,x_{m})$, the corresponding label $Y(y_{1},y_{2},\cdots,y_{m})$ | |
| **Input:** Start bins $n_{1}$, Final bins $n_{2}$ | |
| **Output:** Discretized variables $X_{d}$ | |
| $D(d_{1},d_{2},\cdots,d_{n_{1}})\leftarrow$ discretize X into equal-sized buckets based on sample quantiles; | |
| $T(t_{1},t_{2},\cdots,t_{n_{1}-1})\leftarrow$ compute the $\mathcal{X}^{2}$ value for each pair of adjacent intervals; | |
| **While** $len(T)>n_{2}-1$ **do** | |
|  | $i\leftarrow argmin(T)$; |
|  | delete $t_{i}$ in $T;$ |
|  | $d_{i}\leftarrow d_{i}+d_{i+1}$ (merge the discetization); |
|  | renew the $t_{i-1}$ and $t_{i}$; |
| **end** | |
| return D | |

Two parameters affect this algorithm: the start bins $n_{1}$ determines the computing time and the sensitivity of the algorithm, and the final bins $n_{2}$ influences the subsequent modeling result.

#### 2 Appendix 2. The theory of GBDT

GBDT is a boosting algorithm, combining weak learners to form a strong one. GBDT uses the residual of each predicted result and target value as the target to next learning. The objective function is defined as eq (1), where the regularization term $\Omega$ penalizes the complexity of the model and helps to avoid over-fitting.

$obj(\theta)=\sum_{i=1}^{n} l(y_{i},\hat{y}_{i}^{(t)}+\sum_{k=1}^{K} \Omega(f_{k}))$ (1)

As eq (2)shown, the model trained in additive manner.

$\begin{matrix} obj(\theta) & =\sum_{i=1}^{n} l(y_{i},\hat{y}_{i}^{(t)}+\sum_{k=1}^{K} \Omega(f_{k})) \\ & =\Sigma_{i=1}^{n}l(y_{i},\hat{y}_{i}^{t-1}+f_{t}(x_{i}))+\Omega(f_{k})) \end{matrix}$ (2)

Use second-order approximation to optimize the objective function, and expend $\Omega$ as $\Omega(f)=\gamma T+\frac{1}{2}\lambda w^{2}$ then rewrite the formula as eq (3).

$\begin{matrix} & objj^{t}=\gamma T+\frac{1}{2}\lambda\sum_{j=1}^{T} w_{j}^{2} \\ & +\sum_{i=1}^{n} (l(y_{i},\hat{y}_{i}^{(}t-1))+g_{i}f_{t}(x_{i})+\frac{1}{2}h_{i}f_{t}^{2}(x_{i})) \\ & =\sum_{j=1}^{T} ((\sum_{i\in I_{j}} g_{i})w_{j}+\frac{1}{2}(\sum_{i\in I_{j}} h_{i}+\lambda)w_{j}^{2})+\gamma T \end{matrix}$ (3)

where $g_{i}$ and $h_{i}$ are first and second order gradient statistics in the loss function. Define j as the instance set of leaf. eq (3) can be rewrited as eq (6), just traverse data in different forms.

Define$G_{j}=\sum_{i\in I_{j}} g_{i},H_{j}=\sum_{i\in I_{j}} h_{i}$ and rewrite objective function as eq(4):

$obj^{t}=\sum_{j=1}^{T} \left[ (G_{j}w_{j}+\frac{1}{2}(H_{j}+\lambda)w_{j}^{2} \right]+\gamma T$ (4)

the optimal weight of leaf j ,$w_{j}^{*}$ can be computed by

$w_{j}^{*}=-\frac{G_{j}}{H_{j}+\lambda}=-\frac{1}{H_{j}+\lambda}\times G_{j}$ (5)

and the corresponding optimal value is

$obj^{*}=-\frac{1}{2}\sum_{j=1}^{T} \frac{G_{j}^{2}}{H_{j}+\lambda}+\gamma T$ (6)

This value represents the quality of tree structure and can be used to evaluate decision tree.the information gain after the splitting is

$Gain=\frac{1}{2}(\frac{G_{L}^{2}}{H_{L}+\lambda}+\frac{G_{R}^{2}}{H_{R}+\lambda}-\frac{(G_{L}+G_{R})^{2}}{H_{L}+H_{R}+\lambda})-\gamma$ (7)

This formula is used to evaluate splitting candidates.

Compared to the origin GBDT, Hist-GBDT used many engineering technics to speed up the decision tree construction whereas not reducing the accuracy of prediction. Histogram algorithm is used for feature sorting, which not only reduce consumed time but also have a regularization effect and effectively prevent overfitting. Hist-GBDT also uses leaf-wise generation strategy to reduce training data and uses EFB algorithm to avoid calculation of redundant features.

#### 3 Appendix 3. 10-fold cross validation

The specific steps of 10-fold cross validation are as follows:

**Step 1**: All samples are randomly divided into 10 equal parts to obtain 10 sample subsets.

**Step 2**: 9 sample subsets are selected to form the training set, and the remaining one is the test set. The total score is calculated by Hist-GBDT. 10 models are established after 10 division.

**Step 3**: Test the stability of each model using the test set corresponding to the training set, and define the stability index as follows:

$S_{i}=1-\left| \hat{V_{i}}-V_{i} \right|$ (8)

Where $S_{i}$ is the stability of the i (i = 1,2,……,10) classification system and V is the AUC of the model result on traing dataset and validation dataset.

**Step 4**: The average stability of all models after 10 cross validation is calculated to measure the final stability of the whole system:

$S=\sum_{n=1}^{10} \frac{S_{n}}{10}$ (9)

#### 4 Table 1. Comparison of features between pregnant group and the non-pregnant group

| Variables | Non-pregnancy (-) **(N=20239)** | Pregnancy (+) **(N=16823)** | P-value |
| --- | --- | --- | --- |
| **Data of Man** |  |  |  |
| Age (years) | 33.98±5.94 | 32.61±4.97 | <0.0001 |
| BMI (kg/m^2^) | 24.05±3.51 | 23.98±3.53 | 0.123 |
| Current Smoking | 6129(30.28%) | 5134(30.53%) | 0.626 |
| Normal morphologic rate of sperm (%) | 5.80±10.83 | 5.18±9.46 | <0.0001 |
| **Data of Female** |  |  |  |
| Age (year) | 32.01±4.95 | 30.73±4.08 | <0.0001 |
| BMI (kg/m^2^) | 21.98±2.67 | 21.83±2.62 | <0.0001 |
| Gravidity | 1.12±1.30 | 1.01±1.24 | <0.0001 |
| Parity | 0.20±0.40 | 0.15±0.36 | <0.0001 |
| Basal LH (mIU/ml) | 5.14±2.61 | 5.36±2.77 | <0.0001 |
| Basal FSH (mIU/ml) | 7.05±2.31 | 6.79±2.08 | <0.0001 |
| Basal T (ng/ml) | 0.86±0.62 | 0.87±0.60 | 0.164 |
| Basal P (ng/ml) | 1.71±1.02 | 1.67±0.98 | <0.001 |
| Basal E2 (pg/ml) | 123.97±64.13 | 120.55±60.43 | <0.0001 |
| Basal PRL (ng/ml) | 18.60±9.76 | 19.01±10.01 | <0.0001 |
| AMH (ng/ml) | 3.12±2.56 | 3.63±2.67 | <0.0001 |
| TG (mmol/l) | 1.10±0.55 | 1.08±0.53 | <0.05 |
| LDL-C (mmol/l) | 2.61±0.65 | 2.60±0.65 | 0.089 |
| HDL-C (mmol/l) | 1.37±0.29 | 1.36±0.29 | 0.17 |
| TC (mmol/l) | 4.42±0.76 | 4.39±0.75 | <0.0001 |
| Glucose (mmol/l) | 5.10±0.43 | 5.07±0.42 | <0.0001 |
| AFC(n) | 11.25±2.40 | 12.12±2.33 | <0.0001 |
| History of ART |  |  | <0.0001 |
| ICSI+IVF | 432(2.1%) | 240(1.4%) |  |
| ICSI | 3945(19.15%) | 2670(15.9%) |  |
| IVF | 1556(7.7%) | 1085(6.4%) |  |
| AID or AIH | 1895(9.4%) | 1741(10.3%) |  |
| NA | 12411(61.3%) | 11087(65.9%) |  |
| Type of infertility |  |  | <0.0001 |
| Primary | 11080（ 54.75%） | 8648（ 51.41%） |  |
| Secondary | 8940（ 44.17%） | 7932（ 47.15%） |  |
| Unknown | 219（ 1.08%） | 243（ 1.20%） |  |
| Number of Times about IVF ET | 1.45±0.77 | 1.34±0.67 | <0.0001 |
| Infertility diagnosis |  |  | 0.851 |
| Male | 1814（9.00%） | 1478（8.79%） |  |
| Female | 9292（45.91%） | 7700（45.78%) |  |
| Unexplained | 364（17.99%） | 314（18.66%） |  |
| Multi Causes | 6270（30.98%） | 5200（30.91%） |  |
| Others | 2499（12.34%） | 2131（12.67%） |  |
| Duration of infertility (year) | 3.98±2.94 | 3.61±2.62 | <0.0001 |
| **Data in IVF-ET Procedure** |  |  |  |
| Treatment Strategy |  |  | <0.0001 |
| Long strategy | 8576（ 42.37%） | 8170（ 48.56%） |  |
| Short strategy | 2132（ 10.53%） | 1260（ 7.49%） |  |
| Antagonist strategy | 5150（ 25.45%） | 4444（ 26.42%） |  |
| Ultra-long strategy | 1031（5.10%） | 1105（ 6.57%） |  |
| Microstimulation strategy | 1487（ 7.34%） | 674（ 4.01%） |  |
| Others | 1863(9.21%) | 1170(5.78%) |  |
| Fertilization procedure |  |  | <0.0001 |
| IVF | 13480(66.6%) | 11679(69.4%) |  |
| ICSI | 6453(31.9%) | 4783(28.4%) |  |
| Half ICSI | 303(1.5%) | 357(2.1%) |  |
| Stimulation days (day) | 9.65±2.69 | 10.04±2.41 | <0.0001 |
| Total gonadotropin dose (IU) | 2053.81±790.70 | 2076.47±743.28 | <0.01 |
| Number of Follicles | 10.68±3.82 | 11.90±3.75 | <0.0001 |
| Diameter of The Largest Follicle (cm) | 1.75±0.50 | 1.79±0.46 | <0.0001 |
| Number of Follicles with Diameter larger than 1.4cm | 7.95±2.54 | 8.78±2.62 | <0.0001 |
| FSH before Gonadotropin (mIU/ml) | 5.71±2.86 | 5.33±2.64 | <0.0001 |
| LH before Gonadotropin (mIU/ml) | 3.71±2.42 | 3.69±2.52 | 0.303 |
| E2 before Gonadotropin (pg/ml) | 98.07±86.27 | 89.29±80.18 | <0.0001 |
| LH on day of hCG (mIU/ml) | 3.02±2.58 | 2.70±2.21 | <0.0001 |
| E2 on day of hCG (pg/ml) | 11640.41±8135.42 | 12601.53±8171.93 | <0.0001 |
| P on day of hCG (ng/ml) | 98.07±86.27 | 89.29±80.18 | 0.594 |
| Number of oocytes retrieved | 10.64±6.60 | 11.88±6.51 | <0.0001 |
| Number of 2PN | 5.70±4.15 | 6.69±4.21 | <0.0001 |
| Blastulation (n) | 0.68±1.14 | 0.82±1.26 | <0.0001 |
| Type of endometrial |  |  | 0.078 |
| A | 14691(72.6%) | 12262(72.9%) |  |
| B | 3770(18.6%) | 3146(18.7%) |  |
| C | 239(1.2%) | 231(1.4%) |  |
| Others | 1539(7.6%) | 1184(7.04%) |  |
| Endometrial thickness (cm) | 1.04±0.25 | 1.07±0.25 | <0.0001 |
| Embryo Culture Time (day) | 2.84±0.37 | 2.87±0.34 | <0.0001 |
| Number of Available Embryo | 3.19±2.32 | 3.74±2.42 | <0.0001 |
| Number of embryo transfers | 1.72±0.54 | 1.85±0.48 | <0.0001 |

Data are expressed as mean ± SD or number (percentage). Abbreviations: BMI (body mass index), LH(luteinizing hormone), FSH (follicle-stimulating hormone), T (testosterone), P (progestogen), E2 (estradiol), PRL (prolactin), AMH (anti-Mullerian hormone), TC (total cholesterol), LDL-C (low-density lipoprotein cholesterol), HDL-C (high-density lipoprotein cholesterol) , TG (triglycerides), AFC (Antral follicle count), IVF (in vitro fertilization), ICSI (intracytoplasmic sperm injection), AI (artificial insemination), hCG (human choriogonadotropin).

#### 5 Table 2. The interrelation between age of women and number of cycles in model

| Age (year) | Number of IVF-ET cycles (No.) | Pregnancy rate (%) | Number |
| --- | --- | --- | --- |
| <35 | 1 | 50 | 21296 |
|  | 2 | 45.6 | 5260 |
|  | 3 | 45.5 | 1109 |
|  | 4 | 46.2 | 292 |
|  | ≥5 | 43.4 | 182 |
| ≥35 | 1 | 36.7 | 5235 |
|  | 2 | 32.3 | 2169 |
|  | 3 | 29.2 | 802 |
|  | 4 | 26.4 | 345 |
|  | ≥5 | 23.1 | 372 |

#### 6 Table 3. The interrelation between AMH and COS strategy in model

| AHM (ng/ml) | Treatment Strategy | Pregnancy rate (%) | Number |
| --- | --- | --- | --- |
| ≤1.78 | Long strategy | 47.7 | 488 |
|  | Short strategy | 43.5 | 340 |
|  | Antagonist strategy | 41.9 | 1586 |
|  | Ultra-long strategy | 57.8 | 121 |
|  | Microstimulation strategy | 43.5 | 340 |
|  | Others | 38.3 | 944 |
| >1.78 | Long strategy | 48.8 | 16258 |
|  | Short strategy | 36.4 | 3052 |
|  | Antagonist strategy | 47.2 | 8008 |
|  | Ultra-long strategy | 51.4 | 2015 |
|  | Microstimulation strategy | 30.4 | 1593 |
|  | Others | 38.7 | 2089 |
